# Eviscerated liver: an extremely rare complication of abdominal wound dehiscense through a midline incision

**DOI:** 10.1093/jscr/rjad609

**Published:** 2023-11-10

**Authors:** Mohanad Elbashier, Ahmed Rafei, Abdulwahab Abdulkarim, Mohamed Soud, Hassan Musa, Ali Taher, Alsadig Suliman

**Affiliations:** Department General Surgery, Sudanese Medical Council, Khartoum 11115, Sudan; Department of Surgery, Faculty of Medicine, Omdurman Islamic University, Omdurman 11115, Sudan; Department of Surgery, Faculty of Medicine, Hayatt University College, Khartoum, Sudan; Department of General Surgery, University of Gezira, Wad Madni, Sudan; Department of General & Laparoscopic Surgery, Wad Madni Teaching Hospital, Wad Madni, Sudan; Department of General Surgery, Faculty of Medicine, Sudan International University, Khartoum, Sudan; Department General Surgery, Sudanese Medical Council, Khartoum 11115, Sudan

**Keywords:** liver evisceration, postoperative complication, abdominal wound dehiscense, burst abdomen

## Abstract

Abdominal wound dehiscense, or burst abdomen, is a critical postoperative complication necessitating immediate intervention. We present an extremely rare case of left hepatic lobe evisceration through wound dehiscense in a 65-year-old female receiving palliative care for hypopharyngeal squamous cell carcinoma. The patient’s midline incision that was performed for feeding jejunostomy tube displayed liver protrusion on Day 14 postoperatively. Surgical exploration revealed a healthy liver, prompting reduction and secondary sutures to prevent complications. Abdominal wound dehiscense risk factors, including advanced age, poor nutrition, and medical illness, contribute to its occurrence. Although guidelines for liver evisceration management are lacking, our case emphasizes proper technique, wound care, and nutritional support to aid the healing process and to ensure a better outcome for the patients.

## Introduction

Abdominal wound dehiscense (Burst abdomen) is a critical, challenging, and upsetting postoperative complication that many surgeons encounter. Intervention is necessary right once if there is abdominal wound dehiscense, which may be partial or complete disruption without eviscerating the abdominal contents or with it. While most reported cases of abdominal contents evisceration involve bowel [1–3], in our case, the eviscerated organ was the liver which is an extremely rare.

Although the previous reports of liver herniation present as lump in the epigastric region [4–6], in this case, the left lobe of the liver was eviscerating outside of the wound dehiscense. This is an important distinction, as it could affect the approach to treatment and management.

## Case presentation

A 65-year-old female patient a known case of hypopharangeal squamous cell carcinoma for 1 year and was receiving chemo- and radiotherapy as part of regular follow-up care, the patient was referred to our surgery department presenting with progressive difficulty in swallowing for both solids and liquids over the past 6 months, accompanied by significant weight loss.

Based on the recommendation of the oncology department as part of the palliative care for the patient, an open feeding jejunostomy was performed through a midline incision; closing of the wound was done by mass closure using 2-0 nylon suture material, which was successfully performed without intra-operative or post-operative complications. The patient was then discharged in a stable condition, enabling her to continue her recovery at home.

**Figure 1:**
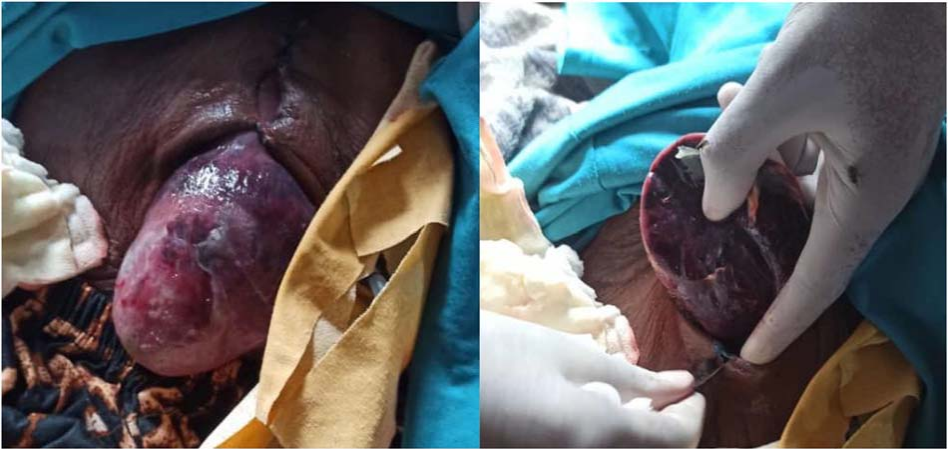
The eviscerated left lobe of the liver through the midline incision.

On Day 14 from the operation, the patient developed wound dehiscense at the surgical site. On physical examination, the patient looks cachectic, the abdomen was distended, a midline incision was visible, extending from the patient’s sternum to the level of the umbilicus, and the left lobe of the liver was protruding through the incision (Fig. 1).

Her vitals were normal. Laboratory results included: hemoglobin: 13.3 g/dl, white blood cell count: 2.7 × 109/L, platelets count: 238 × 109/L, with normal renal and liver function test. Exploration of the wound was done, the liver was examined, revealing no signs of necrosis or tissue damage associated with gangrene. The organ appeared to be in good condition, with normal coloration and texture.

**Figure 2:**
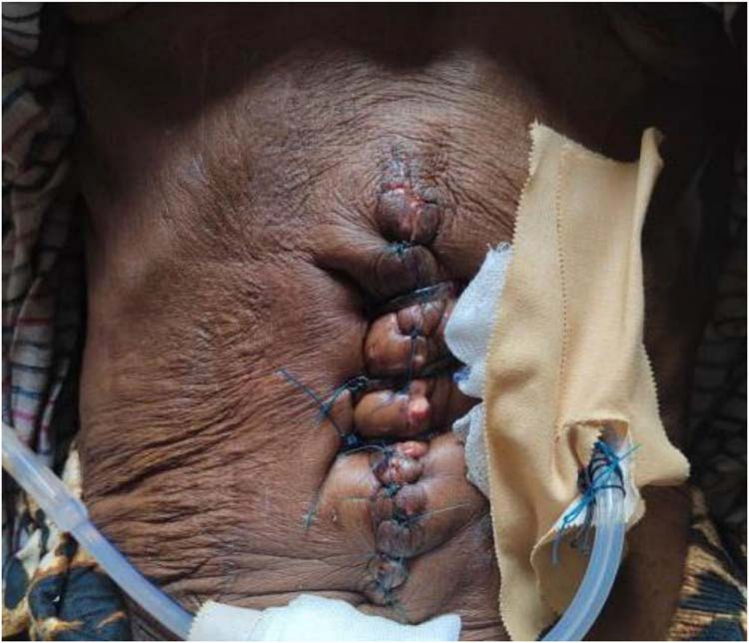
Postoperative image after the secondary tension suture.

Then the patient underwent reduction of the eviscerated liver with secondary tension suture, to ensure proper closure and stability. Then the abdomen was closed using simple sutures and thoroughly washed with povidine-iodine to minimize the risk of infection (Fig. 2).

The patient’s postoperative course was closely monitored to assess her recovery progress. Regular follow-ups were scheduled to evaluate any potential complications or adverse effects resulting from the tension suture procedure.

## Discussion

While liver evisceration is an extremely rare complication of abdominal wound dehiscense, the occurrence of burst abdomen is common. In fact, a study by Talukdar *et al.* [7] was conducted on 217 patients who underwent midline laparotomy. It found out that 12.6% of patients developed wound dehiscense. Furthermore, number of risk factors have been identified as contributing to the development of abdominal wound dehiscense, including advanced age, poor nutrition, and preoperative medical illness including diabetes, anemia, hypoproteinemia, jaundice, renal failure (uremia), cough, peritonitis, malignancy, intra- operative knot breakage, type, and duration of surgery [8].

Additionally, patients are more likely to develop ventral herniation and, as a result, liver evisceration because of weakness in the abdominal wall, increase in intra-abdominal pressure and infection of the wound. Another theory assumed by the Echo *et al*. [9], suggests that possible risk factors include congenital absence of liver’s left and right triangular ligaments. When combined with the aforementioned risk factors, the absence of these structures, which hold the liver to the retroperitoneum, can lead to anterior protruding of liver segments.

However, there are no guidelines or recommendations for the management of this complication, making it impossible to assess the risks and outcomes due to the limited data available and rarity of liver evisceration. This is the only such case and the first case reported from Africa (to the best of our knowledge).

Liver herniation has several complications depending on which lobe of the liver is herniated, left hepatic lobe herniation has been linked to liver incarceration within the hernial sac, which can result in hepatic encephalopathy and liver failure [10]. On the other hand, Budd–Chiari syndrome has been connected to right hepatic lobe herniation. In one instance, a 75-year-old woman who had undergone a right partial nephrectomy 52 years earlier appeared with a right hepatic lobe hepatic lobe herniation. She was asymptomatic but a computed tomography (CT) scan revealed that she had secondary Budd–Chiari syndrome [11]. Liver evisceration is a rarer and less well-understood complications, but it may also have similar consequences.

We performed a reduction of the eviscerated liver to its normal position, and then applied secondary tension sutures to prevent complications like peritonitis, liver trauma, or fluid loss due to the exposure of the liver and evaporation. Moreover, several factors contribute to successful healing in patients with abdominal wound dehiscense. First, proper surgical technique, for patient with nutritional depletion, wound closure with a prophylactic tension suture is recommended to optimize healing, and minimally access surgery for jejunostomy tube is preferred for patients with similar conditions and appropriate wound debridement, is crucial. Second, good wound care, such as keeping the wound clean, can help prevent infection and promote healing. Third, adequate intake of carbohydrates, micronutrients, arginine and glutamine, vitamins (A, B, C, and D), zinc, and iron are essential to aid in the healing process [12].

## Conclusion

Liver evisceration is an extremely rare complication of abdominal wound dehiscense. Proper management and treatment are necessary to prevent further complications. More studies are needed to understand the risk factors and outcomes associated with this complication.

## Data Availability

Any required links or identifiers for the data are present in the manuscript as described. No additional data are available.
